# Body esteem in adolescent hair pullers

**DOI:** 10.1556/JBA.3.2014.010

**Published:** 2014-04-05

**Authors:** ERIN M. ALTENBURGER, ESTHER S. TUNG, NANCY J. KEUTHEN

**Affiliations:** ^1^The Ohio State University, Columbus, OH, USA; ^2^Massachusetts General Hospital/Harvard Medical School, Boston, MA, USA

**Keywords:** hair pulling, trichotillomania, body esteem

## Abstract

*Background and aims:* Trichotillomania (TTM) often first presents in adolescence, a developmental period marked by vulnerability in body image. To date, no one has studied the relationship between this disorder and body esteem. *Methods:* 49 adolescents with DSM-IV TTM or chronic hair pulling (HP) and 23 control adolescents were administered diagnostic assessments and self-report measures of hair pulling and body esteem. *Results:* HP youth vs. controls reported lower levels of body esteem on all Body-Esteem Scale for Adolescents and Adults (BESAA) subscales (appearance, attribution and weight satisfaction). HP contributed to lowered body esteem, independent of comorbid anxiety or depression. As expected, HP youth with vs. without comorbid anxiety or depression reported lowered levels of body esteem. Further, greater HP severity and distress were significantly associated with lower levels of body esteem. HP severity alone but not distress/impairment predicted lower levels of body esteem, independent of comorbid anxiety and depression. *Conclusions:* Both hair pulling and comorbid anxiety and depression can independently impact body esteem in adolescent hair pullers.

## INTRODUCTION

Onset of trichotillomania (TTM) typically occurs in early adolescence (Christenson & Mansueto, 1999), a developmental window characterized by vulnerability in self-esteem and body image (Harper & Marshall, 1991). Relationships between hair pulling (HP) and low self-esteem (Soriano et al., 1996), depressed affect (Keuthen et al., 1998) and shame (Stemberger, Thomas, Mansueto & Carter, 2000) have already been reported for adults.

The relationship between body esteem and HP in adolescents has not yet been empirically studied. Yet, body esteem may be of relevance in adolescents with HP, a disorder associated with potentially disfiguring physical sequelae in multiple body areas (Bohne, Keuthen & Wilhelm, 2005). Further, it is particularly important to study body esteem in this population given the degree of peer pressure, teasing and social comparison surrounding body appearance (e.g. Helfert & Warschburger, 2013).

Although body esteem and self-esteem are closely related, body esteem has multiple domains. Global self-esteem has only been shown to highly correlate with one’s feelings about appearance while thoughts attributed to others regarding body and appearance are unique to body-esteem (Mendelson, White & Mendelson, 1997). Thus, body esteem encompasses more phenomenology than self-esteem and addresses the shame and embarrassment associated with HP. While it is anticipated that HP alone would impact body esteem, it is possible additional psychiatric comorbidities could compound or drive this effect. In comparison to earlier research, our study investigated body vs. self-esteem, focused on youth vs. adult samples, utilized in-person diagnostic assessment vs. self-report, and included a comparison sample.

We had several hypotheses. First, we predicted that youth with HP vs. a comparison sample would report lowered body esteem as reflected in both feelings about their appearance and thoughts attributed to others regarding their body and appearance. While associations have been reported between body esteem and eating disorders, depression and anxiety (e.g. Stice, Hayward, Cameron, Killen & Taylor, 2000; Ivarsson, Svalander, Litlere & Nevonen, 2006; Jónsdóttir, Arnarson & Smári, 2008), we predicted that HP vs. comparison group differences would be maintained independent of depressive and anxiety comorbidity. Secondly, we predicted lower levels of body esteem for HP youth with vs. without depressive or anxiety diagnoses. Thirdly, we hypothesized that greater HP severity and associated distress would be correlated with lower body esteem involving both feelings about their appearance and thoughts attributed to others regarding their body and appearance. Again, we hypothesized that these associations would remain independent of comorbid depressive or anxiety disorders.

## METHODS

### Participants

Our sample consisted of 49 adolescents (13 to 18 years old) with DSM-IV-TR diagnoses of TTM or chronic HP (endorsement of all DSM criteria except B and/or C). Our comparison group consisted of 23 age- and gender-matched adolescents without a diagnosis of TTM or chronic HP. Adolescents with diagnoses of mental retardation, autism spectrum disorders, or psychotic disorders were excluded.

HP youth were predominantly female (*n* = 48) and Caucasian (*n* = 45) with mean (SD) age of 15.07 (1.47) years. In addition, 2.04% (*n* = 1) identified as Black/African-American, 2.04% (*n* =1) as Hispanic/Latino, and 4.08% (*n* =2) as Multi-racial. Comparison adolescents were also predominantly female (*n* = 20) and exclusively Caucasian with mean (SD) age of 15.20 (1.54) years. HP vs. comparison adolescents did not differ significantly (*p*s > .05) on gender, age or ethnicity. Psychiatric comorbidity was compared for HP vs. comparison youth using the Fisher’s exact test; it revealed significant differences on current OCD (HP: *n =* 13, comparison: *n* =1; *X*^2^ = 4.92, *p* = .029), GAD (HP: *n* = 9, comparison: *n* =0; *X*^2^ = 4.83, *p* = .050), and social phobia (HP: *n* =8, comparison: *n* =0; *X*^2^ = 4.22, *p* = .049).

Adolescent hair pullers were recruited from the MGH TTM clinic and the Trichotillomania Learning Center newsletter. The healthy comparison group was recruited from flyers and the MGH intranet. All study participants completed a one-time study visit to assess TTM/chronic HP and other psychiatric disorders. All adolescents were paid for participation.

### >Ethics

Approval was obtained from the Partners Health Care IRB. Study participants completed informed consent prior to participation. This research also complied with the Code of Ethics of the World Medical Association (Declaration of Helsinki).

### Assessment materials

#### Self-report instruments

*Body Esteem Scale for Adolescents and Adults (BESAA)*. The BESAA (Mendelson, Mendelson & White, 2001) is a 23-item measure with three subscale scores. These include subscales measuring general feelings regarding appearance (Appearance; 10 items, e.g. “My looks make me upset”), evaluations attributed to others about one’s body and appearance (Attribution; 5 items, e.g. “Other people consider me good looking”, “I’m as nice looking as most people”) and weight satisfaction (Weight; 8 items, e.g. “I’m proud of my body”). Individuals are asked to rate how often they agree with each item from “never” (1) to “always” (5). Higher subscale scores indicate more positive body esteem; negatively valenced items are reverse-scored. The appearance, weight, and attribution subscales have each shown high internal consistency with Cronbach’s alphas of 0.92, 0.94, and 0.81, respectively. Convergent validity has also been shown through correlation (*r* = .47 to .63) with the Rosenberg Self-Esteem Scale (Mendelson et al., 1997).

*Trichotillomania Scale for Children-Child version (TSC-C).* The TSC-C (Tolin et al., 2008) is a 12-item measure of HP. It consists of severity and distress/impairment subscales. The severity and distress/impairment subscales have each shown good internal consistency with Cronbach’s alphas of 0.76 and 0.84, respectively.

#### Clinician-administered diagnostic measures

*Kiddie-SADS-Present and Lifetime Version (K-SADS-PL)*. The K-SADS-PL (Kaufman et al., 1997) is a semi-structured interview used to assess psychopathology in adolescents using DSM-IV diagnostic criteria.

*Trichotillomania Diagnostic Interview-Revised (TDI-R)*. The TDI-R is a DSM-IV-TR adaptation of the TDI (Rothbaum & Ninan, 1994), a semi-structured interview for TTM diagnosis.

### Statistical analyses

Independent samples *t*-tests were used for group comparisons on all continuous variables. To assess the relationships between continuous variables, Pearson product-moment correlations were used. Multiple regression analyses were used to identify variables predicting body esteem scores.

For purposes of analysis, comorbid anxiety was defined to include OCD, GAD, social phobia, panic disorder with agoraphobia, PTSD and avoidant disorder. Comorbid depression included diagnoses of depression with and without psychotic features. We were unable to include eating disorders in our analyses as only one HP youth had such a diagnosis.

## RESULTS

Do HP vs. comparison youth differ on body esteem?

For our a priori hypothesis regarding the appearance subscale (*g* = 1.04), HP youth (*M* = 18.79, *SD* = 9.04) had significantly lower body esteem scores than comparison youth (*M* = 27.32, *SD* = 5.34; *t*(68) = –4.92, *p* < .001). On the attribution subscale (*g* = .45), HP youth (*M* = 9.71, *SD* = 3.15) had marginally significantly lower scores than comparison youth (*M* = 11.09, *SD* = 2.86; *t*(69) = –1.78, *p =* .080). Exploratory group comparison on the weight subscale (*g* = .55) indicated that HP youth (*M* = 16.07, *SD* = 9.74) also had significantly lower scores than comparison youth (*M* = 21.18, *SD* = 8.15; *t*(64) = –2.12, *p* = .038).

To test our *a priori* hypotheses, multiple linear regression was used to examine the extent to which group condition (HP vs. comparison) and comorbid diagnoses (anxiety or depression) predicted body esteem appearance and attribution scores (Table 1). Assumptions for multiple linear regression were met. Multicollinearity amongst predictors was not found. The total variance in BESAA appearance explained by this model was 33.8% (adjusted *R*^2^ = .32; *F* [2, 67] = 17.12, *p* < .001). Group condition and comorbid anxiety or depression were both statistically significant predictors of BESAA appearance scores. The model predicting BESAA attribution scores was not significant. The exploratory model predicting BESAA weight satisfaction scores was significant and explained 20.2% of the variance in weight satisfaction scores (adjusted *R*^2^ = .18; *F* [2, 63] = 7.97, *p* = .001; Table 1). Only diagnosis of anxiety or depression was significantly predictive of weight satisfaction scores.

**Table 1.** Multiple regression analyses of condition and comorbid diagnoses in predicting BESAA scores^a^

BESAA Appearance				
Predictor variables	B	SE B	β	*p*
Group condition	5.01	2.12	.26	.021^*^
Comorbid anxiety or depression	–7.74	2.06	–.42	<.001^*^
BESAA Weight Satisfaction				
Group condition	1.58	2.49	.08	ns
Comorbid anxiety or depression	–8.18	2.49	–.41	.002^*^

^a^ For group condition, Hair pulling (HP) youth were coded as 1 and control youth were coded as 2.

^*^ *p* < .05, ns = non-significant.

### Do HP youth with and without psychiatric comorbidity differ on body esteem?

HP youth without comorbidity (*n* = 25) scored significantly higher on BESAA appearance (*M* = 23.00, *SD* = 7.58) than HP youth (*n* = 24) with anxiety and depressive comorbidity (*M* = 14.58, *SD* = 8.53; *t*(46) = 3.61, *p* = .001). No significant differences were found for the BESAA attribution subscale. Exploratory group comparison revealed similar findings for weight satisfaction as for the appearance subscale (HP without comorbidity: *M* = 20.43, *SD* = 7.60; HP with comorbidity: *M* = 11.29, *SD* = 9.71; *t*(42) = 3.50, *p* = .001).

### To what extent are HP severity and distress negatively associated with body esteem?

Correlational analyses were used to analyze our *a priori* hypotheses that HP severity and distress would be negatively associated with BESAA attribution and appearance scores. Exploratory analyses were conducted with the weight satisfaction scale. Neither HP severity nor distress was significantly associated with BESAA attribution scores. HP severity and distress/impairment were significantly negatively correlated with BESAA appearance and weight satisfaction scores, such that greater HP severity and distress were associated with lower body esteem regarding both appearance (TSC severity: *r* = –.38, *p* = .009; TSC distress: *r* = –.32, *p* = .031) and weight satisfaction (TSC severity: *r* = –.30, *p* = .052; TSC distress: *r* = –.31, *p* = .044).

Multiple linear regression was used to analyze our *a priori* hypotheses examining the extent to which HP severity or distress and comorbid diagnoses of anxiety or depression predicted body esteem appearance and attribution scores (Table 2). Assumptions for multiple regression were tested and met. Multicollinearity was not found. Separate models were used with HP severity and distress as predictors since they were highly correlated (*r* = .73, *p* < .001). The total variance in BESAA appearance explained by HP severity and comorbid diagnoses was 32.6% (adjusted *R*^2^ = .29; *F* [2, 43] = 10.38, *p* < .001). Both HP severity and comorbid diagnoses were statistically significant predictors of appearance scores. The total variance in BESAA appearance explained by HP distress and comorbid diagnoses was 28% (adjusted *R*^2^ = .25; *F* [2, 43] = 8.38, *p* = .001) with only comorbid diagnoses as a significant predictor. The models predicting BESAA attribution scores were not significant. Exploratory analyses were conducted with the BESAA weight satisfaction scale. The total variance in BESAA weight satisfaction predicted by HP severity and comorbid diagnoses was 31.5% (adjusted *R*^2^ = .28; *F* [2, 39] = 8.97, *p* = .001) with comorbid diagnoses as the only significant predictor though HP severity was a marginally significant predictor. The total variance in BESAA weight satisfaction predicted by HP distress and comorbid diagnoses was 30.5% (adjusted *R*^2^ = .27; *F* [2, 39] = 8.55, *p* = .001). Again only comorbid anxiety or depression was a significant predictor.

**Table 2.** Multiple regression analyses of HP severity/distress and comorbid diagnoses in predicting BESAA scores^a^

BESAA Appearance Model 1				
Predictor variables	B	SE B	β	*p*
HP severity	–5.63	2.35	–.30	.021^*^
Comorbid anxiety or depression	–7.89	2.33	–.43	.001^*^
BESAA Appearance Model 2				
HP distress	–3.68	2.25	–.22	ns
Comorbid anxiety or depression	–7.94	2.43	–.44	.002^*^
BESAA Weight Satisfaction Model 1				
HP severity	–4.61	2.58	–.24	.081
Comorbid anxiety or depression	–9.21	2.58	–.48	.001^*^
BESAA Weight Satisfaction Model 2				
HP distress	–3.82	2.38	–.22	ns
Comorbid anxiety or depression	–8.97	2.63	–.47	.002^*^

^a^ HP = hair pulling.

^*^ *p* < .05, ns = non-significant.

## DISCUSSION

Our results show that body esteem is compromised in all domains (appearance, attribution, and weight) in HP youth when compared to an age- and gender-matched comparison sample. Both comorbid anxiety or depression and group condition significantly predicted feelings regarding appearance. Although diagnosis of anxiety or depression was a stronger predictor, group condition predicted variance in appearance scores independent of comorbid anxiety and depression. Contrary to our predictions, group condition did not significantly predict one’s thoughts attributed to others regarding appearance (attribution subscale) but neither did comorbid diagnoses. Only comorbid anxiety or depression significantly predicted weight satisfaction.

As predicted, HP youth with comorbid anxiety or depression vs. youth with HP alone reported lower levels of body esteem surrounding feelings about appearance. Thus, both HP and comorbidities compound difficulties in body esteem. Although not predicted, HP youth with comorbidity also reported lowered levels of weight satisfaction.

HP severity and distress in hair pullers were associated with lower levels of body esteem surrounding feelings about appearance and weight satisfaction. Similar to our group comparison, no significant correlation was found between HP distress or severity with the attribution subscale. These results were unanticipated given known distress and embarrassment surrounding HP, particularly the concern that sufferers have regarding the thoughts of others.

Follow-up analyses demonstrated that while anxiety and depressive comorbidity play a role, HP severity also predicts body esteem involving feelings surrounding one’s appearance. It is not surprising that HP severity was found to be predictive of the appearance subscale, as this is the subscale most consistently predictive of self-esteem (Mendelson et al., 2001). HP severity measures the urges to pull, frequency of hair pulling, and the amount of hairs pulled. This maps onto the interference and physical impairment resulting from HP. Although HP distress was associated with body esteem, it was not found to be a significant predictor of body esteem scores when comorbid conditions were taken into account. The maintenance of the relationship between body esteem and HP severity but not distress can be explained by the highly physical nature of the disorder.

As seen by our results, body esteem is impaired in hair pullers, which in turn can affect interpersonal functioning and even lead to further psychiatric comorbidities, such as depression and anxiety disorders. Clinical assessment of body esteem levels can be the first step in addressing these difficulties.

Study limitations include lack of assessment for Body Dysmorphic Disorder (BDD, which is not included in the KSADS). While BDD could also impact body esteem, it is unlikely that BDD could explain our results as attribution scale scores did not significantly predict body esteem. Further, we cannot draw definitive conclusions about causality or whether body esteem and HP interact in a bidirectional relationship. It was assumed that the HP and comparison group represented comparable populations as they were both age- and gender-matched. However, future studies would benefit from a larger comparison sample. Further, it is possible that there may be another underlying variable or variables accounting for case vs. comparison group differences and for the relations between hair pulling severity and body esteem.

## CONCLUSIONS

Hair pulling as well as comorbid anxiety and depression can impact body esteem in adolescent hair pullers. Clinicians should assess body esteem in HP youth and provide targeted intervention when needed to preclude compounded difficulties later in development.
